# Effect of diets supplemented with different conjugated linoleic acid (CLA) isomers on protein expression in C57/BL6 mice

**DOI:** 10.1186/s12263-016-0542-2

**Published:** 2016-10-04

**Authors:** L. Della Casa, E. Rossi, C. Romanelli, L. Gibellini, A. Iannone

**Affiliations:** 1 “ProteoWork Lab”, Dipartimento di Medicina Diagnostica, Clinica e di Sanità Pubblica, Università di Modena e Reggio Emilia, via Campi 287, 41125 Modena, Italy; 2 Dipartimento Chirurgico, Medico, Odontoiatrico e di Scienze Morfologiche con Interesse Trapiantologico, Oncologico e di Medicina Rigenerativa, Università di Modena e Reggio Emilia, via Campi 287, 41125 Modena, Italy

## Abstract

**Background:**

The individual genetic variations, as a response to diet, have recently caught the attention of several researchers. In addition, there is also a trend to assume food containing beneficial substances, or to supplement food with specific compounds. Among these, there is the conjugated linoleic acid (CLA), which has been demonstrated to reduce fat mass and to increase lean mass, even though its mechanism of action is still not known. We investigated the effect of CLA isomers (CLA c9,t11 and CLA t10,c12) on the proteomic profile of liver, adipose tissue, and muscle of mouse, with the aim of verifying the presence of a modification in fat and lean mass, and to explore the mechanism of action.

**Methods:**

C57/BL6 mice were fed for 2 months with different diets: (1) standard chow, (2) CLA c9,t11 diet, (3) CLA t10,c11 diet, (4) CLA isomers mixture diet, and (5) linoleic acid diet. The proteomic profile of liver, white adipose tissue, and muscle was investigated. Statistical significance of the spots with an intensity higher than twofold in expression compared to the control was tested using student’s *t* test (two-tail).

**Results:**

We found that both isomers modulate the proteomic profiles of liver, adipose tissue, and muscle by different mechanisms of action. Liver steatosis is mostly due to the isomer CLA t10,c12, since it alters the expression of lipogenetic proteins; it acts also reducing the adipose tissue and increasing fatty acid oxidation in muscle. Conversely, CLA c9,t11 has no relevant effects on liver and adipose tissue, but acts mostly on muscle, where it enhances muscular cell differentiation.

**Conclusions:**

Administration of CLA in humans has to be carefully personalized, since even considering the presence of a species-specific effect, adverse effects might occur on long-term supplementation. Here we demonstrated that, in mouse, CLA is effective in reducing fat mass, but it also induces liver steatosis. The increase of lean mass is linked to an induction of cell proliferation, which, on long-term supplementation, might also lead to adverse effects.

## Background

The individual genetic variations, as a response to diet, have recently caught the attention of several researchers leading to an impressive growth of the nutrigenomic field. The term nutrigenomic, in fact, refers to the associations between specific nutrients and genetic factor and in particular the way in which foods and food ingredients can influence the response to diet. The study of these food-induced modifications may be at the level of single nucleotide polymorphisms rather than at the gene level [6].

In the last 10 years, there is a trend to assume food directly containing beneficial substances or to supplement food with specific compounds that naturally occur in milk and meat and, among these, conjugated linoleic acid (CLA) plays an important role. CLA is a mixture of positional and geometrical isomers of linoleic acid, with two conjugated unsaturated double bonds at various carbon positions in the fatty acid (FA) chain. Two major forms of CLA are CLA c9,t11 and CLA t10,c12, and a mixture of them has been used in most of the published studies. CLA occurs naturally in many foods; however, the most important dietary sources are dairy products and other foods derived from ruminants animals [7]. In fact, CLA originates from two sources: one source is CLA formed during ruminal biohydrogenation of linoleic acid; the other source is CLA synthesized by the animal’s tissues from vaccenic acid, another intermediate in the biohydrogenation of unsaturated fatty acids.

Nowadays, CLA is supplemented in ruminants fodder, not to improve meat quality but to obtain a milk rich in CLA, optimizing the presence of bio-active substances in the lipid fraction. Recently, CLA is also used in gyms as a supplement, in order to quickly obtain a good shape, since it is known that CLA is able to reduce fat body mass, significantly increasing the capacity of resistance to physical effort.

It has been already demonstrated that diet containing CLA reduced the amount of adipose fat in several species including rat, pig, hamster, chicken, and mouse [16]. Several studies suggest that CLA t10,c12 supplementation is able to reduce fat mass in mice and this is associated to a several-fold increase in the amount of fat stored in liver [8, 26].

In the present study, we, as well as others, have found that CLA had a steatosic effect in mice fed with different diets, containing 1 % CLA t10,c12 isomer, 1 % CLA c9,t11 isomer, and a 50:50 mixture of them. None of the published reports has used the proteomic approach to investigate the mechanism of action involved in such lipogenetic/lipolitic effect. We have simultaneously explored the proteome of liver and white adipose tissue (WAT), in order to consider the possibility that a CLA-containing diet may alter lipid metabolism through a modulation of proteins expression.

## Methods

### Animals and feeding protocols

For this study, 25 adult male C57/BL6 mice (Harlan Italy S.r.l., Udine, Italy), weighing 100–120 g, were kept in air-conditioned colony room (temperature 21 ± 1 °C, humidity 50 %) on a natural light-dark cycle and allowed diet and water ad libitum. Housing conditions and experiments were approved by the local Ethical Committee and by the Italian Ministry of Health (prot. # 121, followed by a modification, prot. # 38). The animals were acclimatized to our housing conditions for a week before being fed with different diets. For this purpose, they were divided into five groups receiving the following diets: control group (5 mice) fed with a repelletted purified complete 4RF21 chow; CLA c9,t11 group (5 mice) fed with a 1 % CLA c9,t11 isomer; CLA t10,c12 group (5 mice) fed with a 1 % CLA t10,c12 isomer; CLA mix group (5 mice) fed with a 1 % of a CLA isomer mixture of c9,t11 and t10,c12 (approx. 50:50); and linoleic acid group (5 mice) fed with a 1 % linoleic acid. The dietary treatments were carried out for a 60-day period. All diets were purchased from Mucedola S.r.l., Settimo Milanese, Italy; CLA and CLA isomers were from NV-CHEK-PREP, INC (Elysian, MN, USA). The animals were kept in fasting conditions until the sacrifice. The mice were anesthetized by diethyl ether inhalation and after beheading, liver, muscle, and WAT were collected and immediately frozen in liquid nitrogen for proteomic and western blotting analysis. Additionally, hematoxylin and eosin staining was used to investigate the liver histopathology.

### Proteomic analysis

Proteomic analysis has been performed on liver, muscle, and WAT of all the animals, and each sample was analyzed twice. Tissues (about 150 mg) were crushed to frozen powder by using a pestle under cooling in liquid nitrogen. The powder was incubated for 30 min. at 4 °C in 300 ml of extraction buffer (6 M urea, 2 M thiourea, 4 % CHAPS, 25 mM DTT, 0.2 % ampholytes), containing a protease inhibitor cocktail (Roche Complete EDTA-free, Roche Diagnostic, Milan). Samples were sonicated at 4 °C in rehydratation buffer (10 s/cycle, 3 cycles) for improving cell lysis and then centrifuged at 13,000×*g* for 1 h at 4 °C. The supernatant was collected, and sample concentration was estimated by Bio-Rad Protein Assay (Bio Rad, Hercules, CA, USA).

For isoelectric focusing (IEF), a total of 120 mg of proteins in rehydratation buffer with a final volume of 300 ml and trace of BPB were used in each IPG strips (17 cm, pH 3–10 NL). The strip rehydratation was performed for 12 h at 20 °C with a constant voltage (50 V) in Protean IEF Cell from Bio-Rad, and the isoelectric focusing was carried out by rapidly increasing the voltage until 250 V for 15 min, then linearly increasing the voltage from 250 to 10,000 V for the next 3 h; after that, focusing was continued at 10,000 V until 75,000 V/h and the temperature was maintained at 20 °C. After IEF, the IPG strips were equilibrated in a buffer containing 1 % *m*/*v* DTT by gentle shaking for 15 min. The proceedings were repeated with equilibration buffer containing 2.5 % *m*/*v* iodoacetamide (IAA).

The strips were applied on 12.5 % polyacrylamide gels, and SDS-PAGE was performed in a Protean Plus Dodeca Cell (Bio Rad, Hercules, CA, USA), that guarantees the reliability of data and reduces experimental variation. Indeed, this analysis has been performed in duplicate for each sample type and by the use of a electrophoretic dodeca cell, which allows to run simultaneously 12 gels under identical conditions, reducing the number of run variables and improving reproducibility. The electrophoresis was performed in TGS running buffer (250 mM Tris, 1.92 M glycine, 1 % SDS, pH 8.3) and was carried out with a constant current of 40 mA/gel for 30 min, followed by constant 500 V at 10 °C until BPB dye marker had reached the bottom of the gels. Proteins were visualized with a silver-staining modified protocol compatible with protein digestion and MS analysis (Bellei et al., [3]).

### Image capture and analysis

All stained gel images were captured with a calibrated densitometer GS-800 (Bio Rad, Hercules, CA, USA) and then analyzed with PDQuest 7.3.1, 2D Image Analysis software program (BioRad, Hercules, CA, USA). The quantity of protein in each spot was normalized by the total valid spot intensity according to the manufacturer’s instruction. Only the spots clearly showing a greater than twofold change in expression compared with controls were selected.

### Statistical analysis

Spots with an intensity change higher than twofold in expression compared to the control were undergoing to statistical analysis. Statistical significance was tested using student’s *t* test (two-tail) and the level selected was set at *P* < 0.05. Significantly varied protein spots were purified and sent to *CIGS* (*Centro Interdipartimentale Grandi Strumenti-University of Modena and Reggio Emilia*) for mass spectrometry analysis. Student’s *t* test was also used to evaluate the statistical significance of western blot data.

### Tryptic in-gel digestion of proteins

Spots were excised from gels with a cut end pipette tip and transferred into a microcentrifuge tube (0.5 ml). Briefly, the gel pieces were incubated with 200 ml of 1:1 *v*/*v* solution 30 mM potassium hexacyano-ferrate (III) and 100 mM sodium thiosulphate in order to destain the spots. After two washes with 100 ml of water for 15 min, 100 ml of 100 % acetonitrile was added to restrict the gels. Proteins were reduced and alkylated adding sequentially 50 ml of a DTT solution (10 mM DTT in 25 mM ammonium bicarbonate) and 50 ml of a iodoacetamide solution (55 mM iodoacetamide in 25 mM ammonium bicarbonate). After 15 min in a Savant SpeedVac® concentrator (Thermo Fisher Scientific, USA), a volume of 30 ml trypsin (Promega, Madison WI, USA) solution (12.5 ng/μl in 25 mM ammonium bicarbonate) was added to the spots and the gel pieces were incubated at 4 °C for 30 min. After tryptic digestion, the solution was removed and the samples were incubated at 37 °C o/n in the same solution, without trypsin. Resulting supernatants, representing peptide solution, were recovered and dried in a SpeedVac® concentrator. Finally, 15 ml of a 5 % formic acid solution was added and the mass spectrometry analysis of peptides was performed.

### Mass spectrometry analysis and MS data processing

The spectrometer was an ESI-CHIP 6520 accurate mass Q-TOF-LC/MS (Agilent). Mass spectrometry (MS) data were automatically registered in PKL file format, analyzed, and searched with a mammalian public protein/genome database using MASCOT MS/MS ion search program version 2.2.06 (Matrix Science, http://www.matrixscience.com). Search parameters were set as follows: species rodents; enzyme trypsin; allowance of one missed cleavage site; carbamidomethylation as fixed modification; peptide tolerance ±0.8 Da; MS/MS tolerance error of ±0.4 Da; monoisotopic mass values and protein mass unrestricted. The score cut-off for 95 % protein identification was set to 37. Protein identification was repeated at least once using spots from different gels. The highest score hits among MASCOT search results were selected. The Micromass software (MassLynx™; version 4.1—2005) allows for the automated selection of peptides for fragmentation (and therefore primary structure determination) when peptide ions above a certain detection level are recorded. Since ESI normally produces multiply charged peptide ions, parameters were chosen so that only multiply charged ions were selected for sequencing by MS/MS. The database searched was the SwissProt 2012_07 version (536789 sequences; 190518892 residues).

### Western blot analysis

Equivalent amounts (150 mg) of liver, muscle, and WAT were pulverized using a mortar in liquid nitrogen. The powder was dissolved in 300 ml of extraction buffer (50 mM Tris pH 7.6, 150 mM NaCl, 1 % Triton X-100, 0,1 % SDS, 1 M PMSF) containing a protease inhibitor cocktail (Roche Complete EDTA-free, Roche Diagnostic, Milan) and incubated for 30 min at 4 °C. In order to improve cell lysis, samples were sonicated at 4 °C (10 s/cycle, 3 cycles) and then centrifuged at 13,000×*g* for 1 h at 4 °C. Protein concentration was calculated using Bio-Rad Protein Assay (Bio Rad, Hercules, CA, USA).

A total of 120 mg of proteins in loading buffer 1× (62.5 mM Tris–HCl pH 6.8, 2 % SDS, 10 % glycerol, 0.002 % bromophenol blue) was loaded on 13 % SDS-polyacrylamide gel, and electrophoresis was performed in TGS buffer. After electroblotting, performed at 200 mA for 2 h at 4 °C in transfer buffer 1× (25 mM Tris pH 8.8, 192 mM glycine, 20 % methanol), the proteins were transferred to a nitrocellulose membrane (Trans-Blot Transfer Medium, Bio-Rad). Thus, the membranes were washed in 1× TBST (0.05 M Tris–HCl pH 7.4, 0.15 M NaCl, 0.1 % Tween 20) and blocked in blocking solution (5 % milk in 1 % TBST) for 1 h at room temperature. The proteins blocked on membrane support were then incubated with specific antibodies: polyclonal anti-peroxisomal acyl-coenzime A oxidase 1 (1:1000 dilution o/n, 4 °C, Abcam ab59964), polyclonal anti-ketohexokinase (1:100 diluition for 1 h, 4 °C, Abcam ab38281), polyclonal anti-galectin-3 (1: 300 dilution o/n, 4 °C, Santa Cruz sc-19283), and monoclonal anti-β actin (1:5000 dilution for 2 h, room temperature, Santa Cruz sc-47778) followed by 1 h incubation at room temperature with a secondary specific anti-goat, anti-rabbit, and anti-mouse conjugated to horseradish peroxidase. Anti-peroxisomal acyl-coenzime A oxidase 1 and anti-ketohexokinase were purchased from Abcam (Cambridge, UK), while anti-β actin and anti-galectin-3 were from Santa Cruz Biotechnology (Santa Cruz, CA, USA). Detection was carried out using chemiluminescence blotting substrate (ECL Plus kit, Amersham Bioscences) following the manufacturer’s instructions.

The autoradiographic slabs were captured with a calibrated densitometer GS-800 (Bio Rad, Hercules, CA, USA) and then analyzed with Quantity One software (Bio Rad, Hercules, CA, USA) for densitometric analysis.

### Histological analysis

The liver tissue from different mice group was fixed in 4 % formalin, embedded in paraffin, and cut into 5-micron sections. The sections were stained with Ehrlich’s hematoxylin and eosin (H&E). The results of staining were viewed and photographed with an Nikon Eclips 50i microscope (Nikon. Tokyo, Japan) using a Plan Fluor X lens and a Nikon Digital Sight DS-L1 camera with ×4 and ×40 magnifications. In order to evaluate the steatosis grade, a four-grade semi-quantitative method was used: grade 0, no or minimal steatosis (<5 %); grade 1, ≥5 % but <25 % mild steatosis; grade 2, ≥25 % but <50 % moderate steatosis; and grade 3, ≥50 % severe steatosis.

## Results

### Necroscopic analysis

**Fig. 1:**
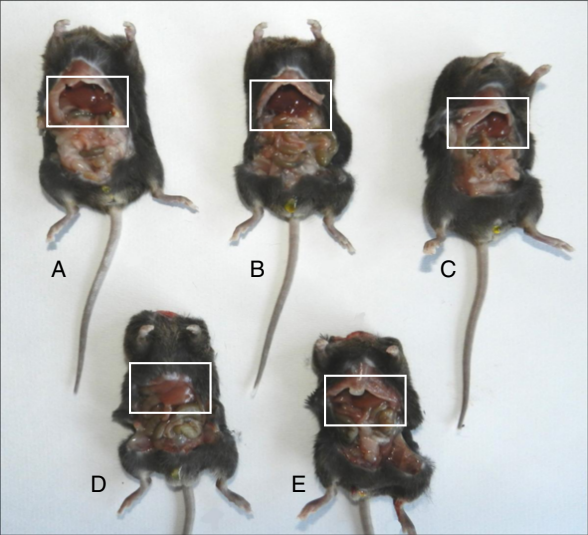
Necroscopic analysis from mice fed with different diets. **a** Control mice. **b** Linoleic acid treated mice. **c** CLA c9,t11 treated mice. **d** CLA t10, c12 treated mice. **e** CLA mix (50:50) treated mice

**Fig. 2:**
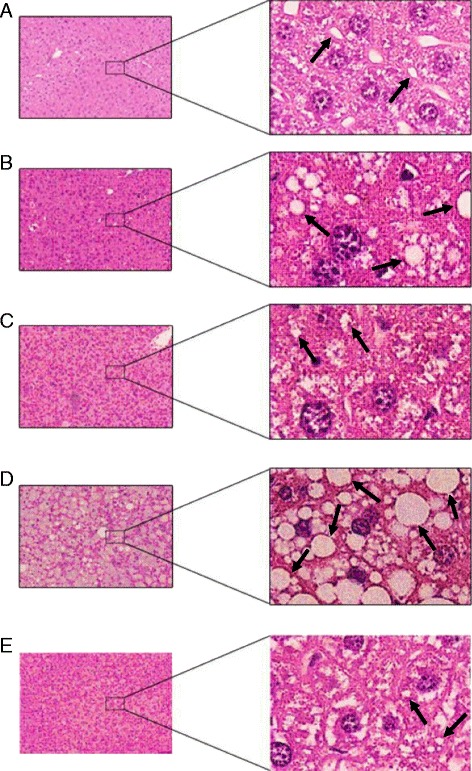
Mice liver sections stained with Ehrlich’s hematoxylin and eosin (H&E). The results of staining were viewed with ×4 and ×40 magnifications. *Arrows* indicate some lipid droplets. Control group fed with a repelletted standard chow (**a**). CLA mix group fed with a 1 % by weight of a mixture of CLA c9,t11 and CLA t10, c12 isomers (**b**). CLA c9,t11 group fed with a 1 % by weight of CLA c9,t11 isomer (**c**). CLA t10, c12 group fed with a 1 % by weight of CLA t10, c12 isomer (**d**). Linoleic acid group fed with a 1 % by weight of linoleic acid (**e**)

**Fig. 3:**
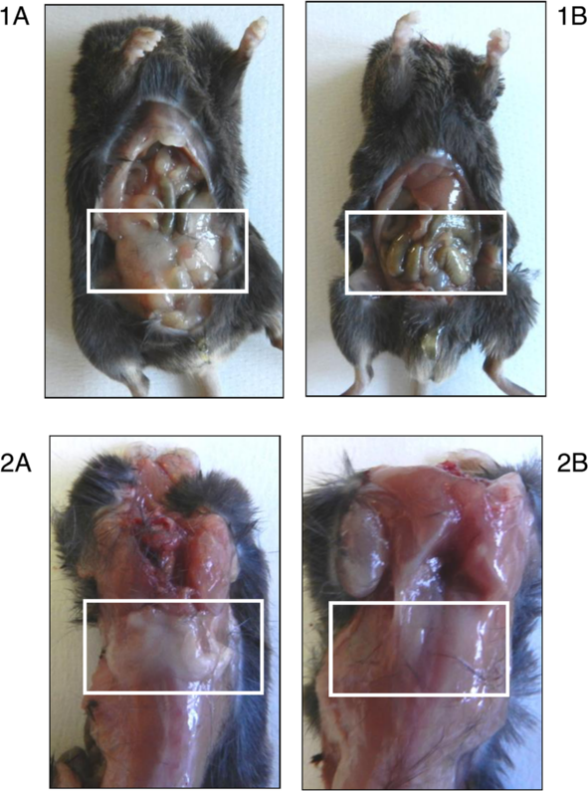
Effect of CLA t10, c12 on white and brown adipose tissue. White adipose tissue (WAT) (1) and brown adipose tissue (BAT) (2) in mice fed with control diet (**1A**, **1B**) and fed with CLA t10, c12 isomer (**2A**, **2B**). Both WAT than BAT completely disappeared in mice fed with CLA t10, c12 compared with mice fed with control diet

After animal sacrifice, the necroscopic analysis showed that livers from CLA-fed mice were enlarged for fat accumulation (Fig. 1) and the degree of this phenomenon was different depending on the type of diet. Steatosis was mild in mice fed with CLA c9,t11 diet, moderate in mice fed with CLA mix diet, and severe in mice fed with CLA t10,c12 diet: this latter was also pale in comparison to all the other livers. This result was supported by the histological analysis (Fig. 2). Furthermore, in mice fed with CLA t10,c12, both white adipose tissue (WAT) and brown adipose tissue (BAT) completely disappeared as shown in Fig. 3.

### Liver proteomic analysis

**Fig. 4:**
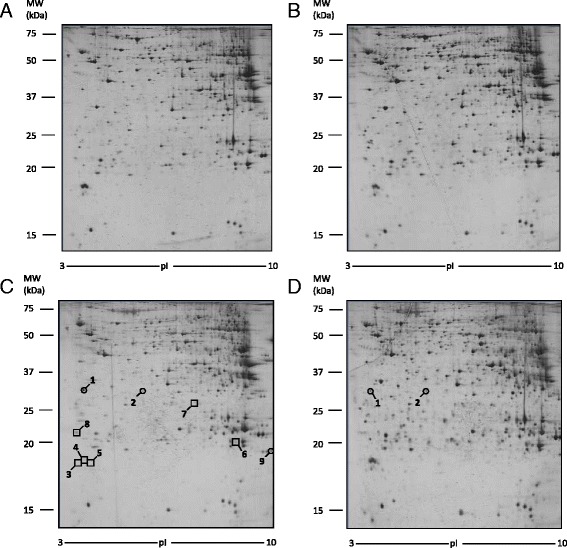
Differentially expressed proteins in liver tissue. Spot number reflects the number reported in Table 1. Molecular weight (KDa) and isoelectric value (pI) are show on the image. *Circles* indicate proteins up-regulated while *squares* indicate proteins down-regulated in CLA treated sample with respect to control sample. **a** Control mice. **b** CLA c9,t11 treated mice. **c** CLA t10, c12 treated mice. **d** CLA mix (50:50) treated mice

To evaluate whether the diet with different CLA isomers could modify proteins expression in liver tissue, we performed 2-DE analysis. Liver samples from mice treated with oleic acid were used as control, since no difference was found in proteomic profile between mice fed with oleic acid and mice fed with a standard chow. The analysis of control samples revealed a number of 900 spots on each gel, 450 of which were selected for PDQuest analysis (Fig. 4). Only the spots that were in accordance with the selection criterion (± twofold change) and statistically significant were excised and subjected to ESI-Q-TOF-MS/MS. All identified proteins are reported in Table 1. As shown in Fig. 4, in liver of mice treated with CLA t10,c12, a modification of nine spots was observed with respect to the control group. In particular, we found the down-regulation of six proteins: major urinary protein 6 (MUP6, spot # 3), major urinary protein 2 (MUP2, spot # 4 and 5), glutathione S-transferase P1 (GSTP1, spot # 6), bile salt sulfotransferase 2 (ST2A2, spot # 7), alanine-TRNA ligase, and cytoplasmic (SYAC, spot # 8). Three proteins have been found up-regulated: annexin A5 (ANXA5, spot # 1), ketoexokinase (KHK, spot # 2), and peroxisomal acyl-coenzyme A oxidase 1 (ACOX1, spot # 9). Moreover, two of these proteins, ANXA5 and KHK, were also up-regulated in the liver of mice fed with CLA mix.

**Table 1:** Proteins separated by 2-DE electrophoresis and identified by MS in liver tissue

Spot^a^	Identification (Swiss-Prot)^b^	Gene^c^	Sequence^c^ coverage (%)	Spot^d^ MW (KDa)	Spot^e^ pI	Score^f^	Swiss^g^ Prot access #
1	Annexin A5 (ANXA5)	Anxa5	72	35.8	4.8	631	P48036
2	Ketohexokinase (KHK)	Khk	61	32.8	5.8	475	P97328
3	Major urinary protein 6 (MUP6)	Mup6	52	20.7	4.7	284	P02762
4	Major urinary protein 2 (MUP2)	Mup2	73	20.7	4.9	227	P11589
5	Major urinary protein 2 (MUP2)	Mup2	51	20.7	4.9	96	P11589
6	Glutathione S-transferase P1 (GSTP1)	Gstp1	39	20.6	8.1	211	P19157
7	Bile salt sulfotransferase 2 (ST2A2)	Sult2a2	10	33.3	6.9	45	P50236
8	Alanine-TRNA ligase, cytoplasmic (SYAC)	Aars	1	106.9	5.4	34	Q8BGQ7
9	Peroxisomal acyl-coenzyme A oxidase 1 (ACOX1)	Acox1	15	74.7	8.6	177	Q9R0H0

^a^2-DE gel image spot number presented in Fig. 1

^b^Commonly used protein name

^c^Gene symbol

^d^Percentage of amino acid sequences for the identified protein

^e^MW and pI = molecular weight and isoelectric point of the protein

^f^Score = [−10 log (*P*)] and *P* is the absolute probability that the observed match between the experimental data and the database sequence is a random event, obtained with MASCOT for all proteins listed

^g^Swiss-Prot primary protein accession number

**Fig. 5:**
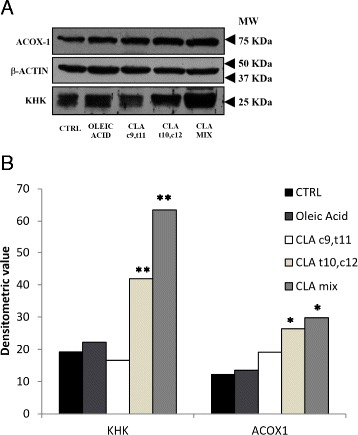
Western blot analysis of liver lysates. **a** Ketoexokinase and peroxisomal acyl-coezyme A oxidase 1 expression were detected by anti-KHK and anti-ACOX1 antibody, respectively. β-actin level was measured as loading control. **b** Densitometric analysis of KHK and ACOX1 levels; densitometric values were normalized by the corresponding value for β-actin. (*p < 0.05; **p < 0.01)

In order to confirm the result obtained by 2-DE analysis, we performed a western blot analysis using anti-ACOX1 and anti-KHK antibodies. As illustrated in Fig. 5, the expression of ACOX1 and KHK normalized by corresponding ß-actin was significantly increased in mice treated with CLA mix and CLA t10,c12.

### Adipose tissue proteomic analysis

**Fig. 6:**
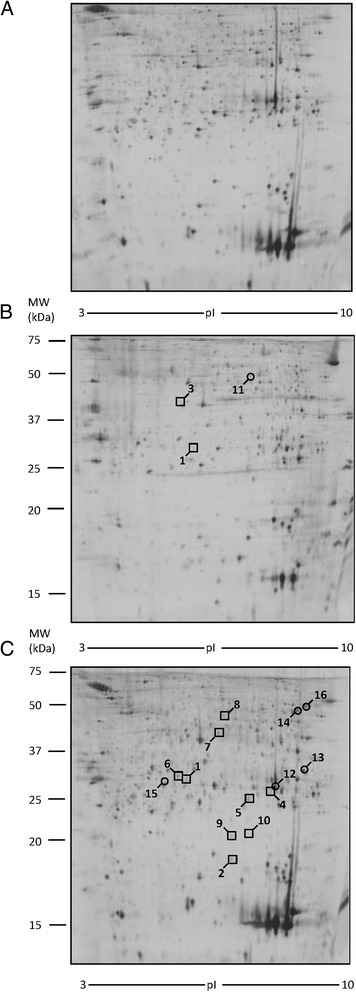
2-DE images of proteins differentially expressed in white adipose tissue (WAT). Spot number reflects the number reported in Table 2. Molecular weight (KDa) and isoelectric value (pI) are show on the image. *Circles* indicate proteins up-regulated, while *squares* indicate proteins down-regulated in CLA treated sample with respect to control sample. **a** Control mice. **b** CLA c9,t11 treated mice. **c** CLA mix (50:50) treated mice

The study of proteomic profile of WAT extracts was performed using the same approach used for liver tissue (2-DE, spectrometry mass analysis and western blot confirmation). This analysis was not carried out in mice fed with CLA t10,c12, since no trace of adipose tissue was found in these mice. Also in this tissue, no differences were found between WAT proteomic profile from mice fed with oleic acid and mice fed with control diet: as a consequence, WAT samples from mice treated with oleic acid were used as control. The analysis of control samples revealed a number of 400 spots on each gel, 250 of which were selected for PDQuest analysis (Fig. 6). Only the spots that were in accordance with the selection criterion (± twofold change) and statistically significant were excised and subjected to ESI-Q-TOF-MS/MS (Table 2). Figure 6 shows that CLA mix treatment causes the down-regulation of nine proteins: indolethylamine *N*-methyltransferase (INMT, spot #1), destrin (DEST, spot # 2), glutathione S transferase theta-1 (GSTT1, spot # 4), mitochondrial peptide methionine sulfoxide reductase (MSRA, spot # 5), endoplasmic reticulum resistant protein 29 (ERP29, spot # 6), delta-amino levulinic acid dehydratase (HEM2, spot # 7), actin, alpha cardiac muscle 1 (ACTC, spot # 8), histone H2B type 1-B (H2B1B, spot # 9), and peptidyl prolyl cistrans isomerase C (PPIC, spot # 10). Five proteins were up-regulated: triosephosphate isomerase (TPIS, spot #12), galectin 3 (LEG-3 spot # 13), citrate synthase, mitochondrial (CISY, spot # 14), AP-5 complex subunit beta 1 (AP5B1, spot # 15), and elongation factor 1 alpha-1 (EF1A1, spot #16). Mice fed with a CLA c9,t11 isomer diet showed a down-regulation of indolethylamine *N*-methyltransferase (INMT, spot #1) and pyridoxal kinase (PDXK, spot #3) and an up-regulation of 2-amino-3-ketobutyrate coenzyme A ligase (KBL, spot # 11).

**Table 2:** Proteins separated by 2-DE electrophoresis and identified by MS in adipose tissue

Spot^a^	Identification (Swiss-Prot)^b^	Gene^c^	Sequence^c^ coverage (%)	Spot^d^ MW (KDa)	Spot^e^ pI	Score^f^	Swiss^g^ Prot access #
1	Indolethylamine *N*-methyltransferase (INMT)	iNMT	40	29.5	6.0	282	P40936
2	Destrin (DEST)	DSTN	41	18.4	8.2	126	Q9R0P5
3	Pyridoxal kinase (PDXK)	Pdxk	26	35.0	5.9	116	Q8K183
4	Glutathione S transferase theta-1 (GSTT1)	GSTT1	32	27.2	7.2	181	P30711
5	Mitochondrial peptide methionine sulfoxide reductase (MSRA)	Msra	19	23.8	6.7	27	Q9D6Y7
6	Endoplasmic reticulum resistant protein 29 (ERP29)	Erp29	29	25.7	5.7	185	P57759
7	Delta-amino levulinic acid dehydratase (HEM2)	Alad	20	36.0	6.3	263	P10518
8	Actin, alpha cardiac muscle 1 (ACTC)	Actc 1	38	41.8	5.2	606	P68033
9	Histone H2B type 1-B (H2B1B)	Hist1h2bb	27	13.8	10.3	67	Q64475
10	Peptidyl prolyl cistrans isomerase C (PPIC)	Ppic	14	22.8	7.0	172	P30412
11	2 Amino 3 ketobutyrate CoA ligase (KBL)	Goat	39	42.8	6.5	2133	O88986
12	Triosephosphate isomerase (TPIS)	Tip1	70	32.2	5.6	1581	P17751
13	Galectin 3 (LEG3)	Lgals3	29	27.6	8.5	500	P16110
14	Citrate synthase mitochondrial (CISY)	Cs	26	49.0	8.2	293	Q9CZU6
15	AP-5 complex subunit beta 1 (AP5B1)	Ap5b1	1	94.0	5.7	22	Q3TAP4
16	Elongation factor 1 alpha-1 (EF1A1)	Ef1a1	29	50.1	9.1	500	P10126

^a^2-DE gel image spot number presented in Fig. 1

^b^Commonly used protein name

^c^Gene symbol

^d^Percentage of amino acid sequences for the identified protein

^e^MW and pI = molecular weight and isoelectric point of the protein

^f^Score = [−10 log (*P*)] and *P* is the absolute probability that the observed match between the experimental data and the database sequence is a random event, obtained with MASCOT for all proteins listed

^g^Swiss-Prot primary protein accession number

**Fig. 7:**
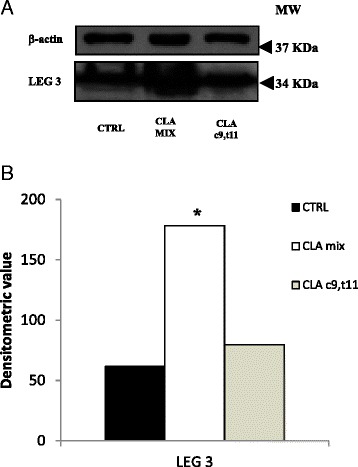
Western blot analysis of adipose tissue lysates. **a** Galectin 3 expression was detected by anti-LEG-3 antibody. β-actin level was measured as loading control. **b** Densitometric analysis of galectin 3 level; densitometric value was normalized by the corresponding value for β-actin. (*p < 0.05)

An anti-LEG-3 antibody was used for western blot analysis in order to confirm the 2-DE results. As shown in Fig. 7, the expression of LEG-3, normalized by corresponding β-actin, was significantly increased in mice treated with CLA mix.

### Muscle tissue proteomic analysis

**Fig. 8:**
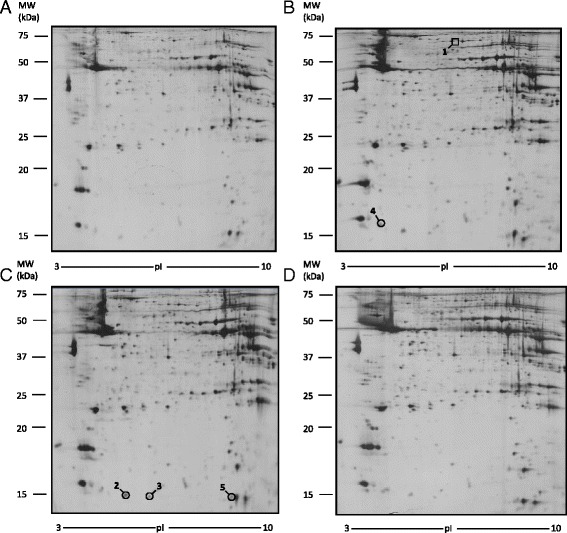
Differentially expressed proteins in muscle tissue. Spot number reflects the number reported in Table 3. Molecular weight (KDa) and isoelectric value (pI) are show on the image. *Circles* indicate proteins up-regulated, while a square indicates proteins down-regulated in CLA treated sample with respect to control sample. **a** Control mice. **b** CLA c9,t11 treated mice. **c** CLA t10, c12 treated mice. **d** CLA mix (50:50) treated mice

Extracts from muscle tissue were processed for proteomic analysis, mass spectrometry analysis, and finally for western blot analysis, to confirm the results. Since no differences were found between muscle proteomic profile from mice fed with oleic acid and mice fed with control diet, muscle samples from mice treated with oleic acid were used as control. In each gel, 700 spots were revealed and 420 of these selected for PDQuest analysis. Only the spots statistically different from control were excised and subjected to ESI-Q-TOF-MS/MS. Results from the MS analysis are reported in Table 3. CLA c9,t11 treatment modified the expression of two proteins, phosphoglucomutase-1 (PGM1, spot # 1) that was down-regulated and galectin-1 (LEG1, spot # 4) that was up-regulated. Furthermore, the proteomic profile of mice fed with CLA t10,c12 shows the up-regulation of three proteins: two isoforms of fatty acid-binding protein, heart (FABPH, spots # 2 and 3) and fumarate hydratatase, mitochondrial (FUMH, spot # 5). The different protein expressions are also indicated in Fig. 8.

**Table 3:** Proteins separated by 2-DE electrophoresis and identified by MS in muscle tissue

Spot^a^	Identification (Swiss-Prot)^b^	Gene^c^	Sequence^c^ coverage (%)	Spot^d^ MW (KDa)	Spot^e^ pI	Score^f^	Swiss^g^ Prot access #
1	Phosphoglucomutase-1 (PGM1)	Pgm1	45	61.7	6.1	816	Q9DOF7
2	Fatty acid-binding protein, heart (FABPH)	Fabp3	59	14.8	6.1	790	P11404
3	Fatty acid-binding protein, heart (FABPH)	Fabp3	66	14.8	6.1	533	P11404
4	Galectin-1 (LEG1)	Lgals1	31	15.2	5.3	135	P16045
5	Fumarate hydratase, mitochondrial (FUMH)	Fh	1	54.5	9.1	15	P97807

^a^2-DE gel image spot number presented in Fig. 1

^b^Commonly used protein name

^c^Gene symbol

^d^Percentage of amino acid sequences for the identified protein

^e^MW and pI = molecular weight and isoelectric point of the protein

^f^Score = [−10 log (*P*)] and *P* is the absolute probability that the observed match between the experimental data and the database sequence is a random event, obtained with MASCOT for all proteins listed

^g^Swiss-Prot primary protein accession number

**Fig. 9:**
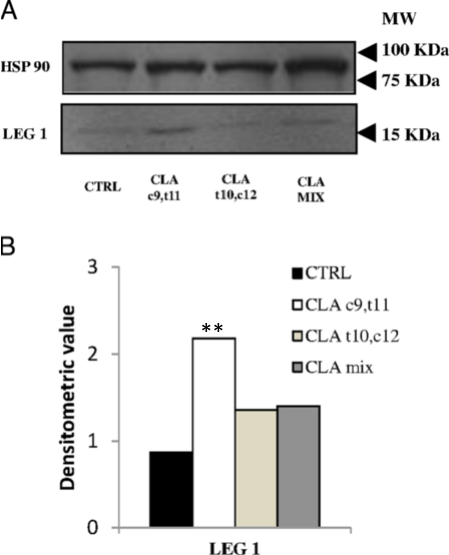
Western blot analysis of muscle tissue lysates. **a** Galectin 1 expression was detected by anti-LEG-1 antibody. HSP90 level was measured as loading control. **b** Densitometric analysis of galectin 1 level; densitometric value was normalized by the corresponding value for HSP90. (**p < 0.01)

The western blot analysis, performed with an anti-LEG1 antibody, confirmed these results (Fig. 9).

## Discussion

In the present study, we evaluated the proteomic profile of liver and WAT, in order to identify a proteomic pattern related to modifications that occurred after the administration of diet supplemented with different CLA isomers. We found differential effects using the separate isomers in comparison to the mixture of them: in this case, we have to take into account that in the mixture the amount of the individual isomers is the half, being the final concentration always 1 %. For interpretation of data obtained through proteomic analysis, we categorized all identified proteins into three groups: proteins disregulated in liver tissue, proteins disregulated in WAT, and proteins disregulated in muscle.

### Proteins disregulated in the liver tissue

We found an up-regulation of ketohexokinase (KHK) in mice fed with CLA t10,c12 isomer. Fructose is metabolized in the liver by fructokinase (KHK), which uses ATP to phosphorylate fructose to fructose-1-phosphate. Fructokinase exists in two isoforms: fructokinase C and fructokinase A, where fructokinase C is the principal isoform in liver and rapidly leads to an intracellular ATP depletion; fructokinase A, instead, metabolizes fructose slowly, without significant ATP consumption [15]. The metabolism of fructose results in nucleotide turnover and uric acid generation that may have a role in inducing mitochondrial oxidative stress and fat accumulation [20, 21]. The up-regulation of KHK was demonstrated in mice fed with a high-fat diet and correlates with steatosis and obesity: for this reason, it has been proposed as an early marker of obesity [25]. Another study shows that the metabolism of fructose by KHK results in intracellular production of ureic acid and that its blockade in intracellular compartment could inhibit the increase in fat accumulation due to fructose [22].

In animal cells, mitochondria, as well as peroxisomes, oxidizes long chain fatty acids via β-oxidation [28]. When cytosolic fatty acids accumulate as a consequence of an impairment of oxidative capacity in mitochondria, alternative pathways in the peroxisomes (β-oxidation) and in microsomes (ω-oxidation) are activated. In peroxisomal β-oxidation, ACOX-1 catalyzes the initial oxidation of very long fatty acids to acyl-CoA. In non-alcoholic fatty liver disease (NAFLD), where a triglyceride accumulation in hepatocytes (hepatic steatosis) is present, Kohjima et al. demonstrated that the expression of ACOX-1 was increased twofold compared with that in the normal liver [19]. The up-regulation of ACOX-1 with CLA t10,c12 isomer that we observed suggested an increase in fatty acid β-oxidation in peroxisomes.

The increase of ACOX-1 by dietary CLA has been previously described by Belury et al. [4] as a result of its activity on PPAR alpha [23], and in addition, it has been shown that it undergoes peroxisomal beta oxidation probably inducing PPAR alpha [1] and may interfere with other peroxisomal beta oxidation substrate [14].

Major urinary protein (MUP) family members are secreted proteins produced predominantly from the liver and excreted into urine [9, 30]. Is well known that MUPs carry small hydrophobic ligands such as pheromones [13, 33], while their endocrine role is less clear. Scientists found that in addition to mediating chemical signaling, circulatory MUP1 regulates glucose and lipid metabolism in animals. Indeed, overexpression of MUP1 suppresses the expression of both glucogenic and lipogenic genes in liver [12, 35]. The role of the other MUPs member is not well documented and therefore requires further studies. In our experiments we found, in mice fed with CLA t10,c12, the down-regulation of other two members of MUP family, MUP2 and MUP6. None of the published reports highlights the effects of MUP2 and MUP6 on lipidic and glucidic metabolism; for this reason, we suggest that a down-regulation of MUP2 and MUP6 may have a role in the genesis of steatosis and they could be considered as markers of steatosis.

It has to be mentioned that liver steatosis by dietary CLA seems to be restricted to mice, since in models of obesity in rats, such as the Zucker, that develop fatty liver, CLA has actually an opposite effect [24], probably mediated by PPAR alpha-mediated activity [27].

### Proteins disregulated in WAT

In WAT tissue, only the CLA mix treatment caused the up-regulation of three proteins related to lipid metabolisms: triosephosphate isomerase (TPIS), citrate synthase, mitochondrial (CISY), and galectin 3 (LEG-3). We suppose that the increased expression of these three proteins was determined by the effect of CLA t10,c12 isomer, since the CLA c9,t11 isomer did not modify the proteomic profile of WAT tissue.

The up-regulation of TPIS was demonstrated in rat fed with high-fat lipid diet by Saggerson [29]. In our experiments, we found the up-regulation of this protein in mice fed with CLA mix.

The acetyl-CoA, in order to be converted into fatty acids, must leave the mitochondria as citrate, and this reaction is operated by the CISY. Therefore, the citrate is cleaved into acetyl-CoA and oxaloacetate. Dietary supplementation with long-chain fatty monounsaturated fatty acids significantly increases the CISY expression [34]. The up-regulation of CISY, in CLA mix treated mice, that we observed explains the increased acetyl-CoA bioavailability for fatty acids synthesis, and therefore, this protein can be considered as a possible marker of accumulation of fatty acids.

Galectin-3 is a 30-KDa lectin which has a C-terminal carbohydrate-recognition domain and an N-terminal domain comprising multiple repeat sequences. Kiwaki et al. demonstrated that adipose tissue synthesized galectin-3 and that the up-regulation of this protein might play a role in adipose tissue growth induced by high-fat diets [18]. In our experiments, we found the up-regulation of this protein in mice fed with CLA mix.

### Proteins disregulated in muscle

In muscle tissues analyzed, we found a disregulation of three proteins: fatty acid-binding protein, heart (FABPH), fumarate hydratase mitochondrial (FUMH), and galectin-1 (LEG1).

More in detail, FABPH is a cytosolic protein belonging to the fatty acid-binding proteins family (FABPs), small ubiquitous proteins (~15 KDa) that have an high affinity with very long chain fatty acid. FABPH is expressed in cardiac and muscular tissue and it is involved in the mitochondrial β-oxidation as very long chain fatty acids and acyl-CoA intracellular transporter. Tan et al. [32] demonstrated that FABPH may also interact with PPARα, nuclear receptors that induced mitochondrial and peroxisomal β-oxidation. The observed up-regulation of FABPH with CLA t10,c12 isomer suggests an increase in fatty acid β-oxidation in peroxisomes.

CLA t10,c12 induces also the up-regulation of FUMH, an enzyme involved in Krebs cycles that converts fumarate to malate: this could indicate an increase of lipid catabolism and a greater ATP availability in muscle tissues.

LEG1 belongs to a family of carbohydrates binding protein and it is primarily expressed in skeletal muscle [2]. Goldring et al. demonstrated that the addiction of LEG1 to cultures of dermal fibroblasts can induce their conversion into muscle cells [10, 11] and the presence of galectin-1 in cultured medium of mesenchymal human stem cells increases than 30 % the cells differentiation [31]. In our experiments, the up-regulation of LEG1 by CLA c9,t11 treatment could therefore indicate an enhancement in muscular cells differentiation, leading to an increase in lean body mass. Moreover, these data may have an important implication regarding muscle tissue repair after exercise [5]. It may also explain the effect of CLA on skeletal muscle metabolism as recently reviewed [17].

## Conclusions

The mechanism of steatogenic action of CLA is clearly mediated by the increased expression of lipogenetic proteins: the up-regulation of ketohexokinase correlates with steatosis and obesity, while the up-regulation of the peroxisomal acyl-coenzyme A oxidase-1 suggests an increase in fatty acid β-oxidation in peroxisomes. The down-regulation of the major urinary proteins could be responsible for a PPAR-γ mediated lipogenesis. The two CLA isomers have different steatogenic effect, being the most evident effect due to the CLA t10, c12 isomer. Being both the tested isomers present in commercially available CLA mixture, we suggest caution in using it as a supplement in human diet. The effect on body lean mass seems to be exclusively due to the isomer CLA c9,t11.

## Abbreviations

ACOX1: Peroxisomal acyl-coenzyme A oxidase 1
ACTC: Actin, alpha cardiac muscle
ANXA5: Annexin A5
AP5B1: AP-5 complex subunit beta 1
BAT: Brown adipose tissue
CISY: Citrate synthase, mitochondrial
CLA: Conjugated linoleic acid
DEST: Destrin
EF1A1: Elongation factor 1 alpha-1
ERP29: Endoplasmic reticulum resistant protein 29
FABPH: Fatty acid-binding protein, heart
FUMH: Fumarate hydratatase, mitochondrial
GSTP1: Glutathione S-transferase P1
GSTT1: Glutathione S transferase theta-1
H2B1B: Histone H2B type 1-B
HEM2: Delta-amino levulinic acid dehydratase
INMT: Indolethylamine *N*-methyltransferase
KBL: 2-Amino-3-ketobutyrate coenzyme A ligase
KHK: Ketoexokinase
LEG: Galectin
MSRA: Mitochondrial peptide methionine sulfoxide reductase
MUP: Major urinary protein
PDXK: Pyridoxal kinase
PGM1: Phosphoglucomutase-1
PPIC: Peptidyl prolyl cistrans isomerase C
ST2A2: Bile salt sulfotransferase 2
SYAC: Alanine-TRNA ligase, cytoplasmic
TPIS: Triosephosphate isomerase
TRNA ligase: Cytoplasmic
WAT: White adipose tissue

## References

[1] Banni S, Petroni A, Blasevich M, Carta G, Angioni E, Murru E, Day BW, Melis MP, Spada S, Ip C (2004). Detection of conjugated C16 PUFAs in rat tissues as possible partial beta-oxidation products of naturally occurring conjugated linoleic acid and its metabolites. Biochim Biophys Acta.

[2] Barondes SH, Cooper DN, Gitt MA, Leffler H (1994). Galectins. Structure and function of a large family of animal lectins. J Biol Chem.

[3] Bellei E, Rossi E, Lucchi L, Uggeri S, Albertazzi A, Tomasi A, Iannone A (2008). Proteomic analysis of early urinary biomarkers of renal changes in type 2 diabetic patients. Proteomics Clin. Appl.

[4] Belury MA, Moya-Camarena SY, Liu K-L, Vanden Heuvel JP (1997). Dietary conjugated linoleic acid induces peroxisome-specific enzyme accumulation and ornithine decarboxylase activity in mouse liver. J Nutr Biochem.

[5] Cerri DG, Rodrigues LC, Stowell SR, Araujo DD, Coelho MC, Oliveira SR, Bizario JC, Cummings RD, Dias-Baruffi M, Costa MC (2008). Degeneration of dystrophic or injured skeletal muscles induces high expression of galectin-1. Glycobiology.

[6] Chadwick R (2004). Nutrigenomics, individualism and public health. Proc Nutr Soc.

[7] Chin SF, Liu W, Storkson JM, Ha YL, Pariza MW (1992). Dietary sources of conjugated dienoic isomers of linoleic acids, a newly recognized class of anticarcinogens. J Fodd Compons Anal.

[8] Clement L, Poirier H, Niot I, Bocher V, Guerre-Millo M, Krief S, Staels B, Besnard P (2002). Dietary trans-10, cis-12 conjugated linoleic acid induces hyperinsulinemia and fatty liver in the mouse. J Lipid Res.

[9] Finlayson JS, Asofsky R, Potter M, Runner CC (1965). Major urinary protein complex of normal mice: origin. Science.

[10] Goldring K, Jones GE, Thiagarajah R, Watt DJ (2002). The effect of galectin-1 on the differentiation of fibroblasts and myoblasts in vitro. J Cell Sci.

[11] Goldring K, Jones GE, Watt DJ (2000). A factor implicated in the myogenic conversion of nonmuscle cells derived from the mouse dermis. Cell Transplant.

[12] Hui X, Zhu W, Wang Y, Lam KS, Zhang J, Wu D, Kraegen EW, Li Y, Xu A (2009). Major urinary protein-1 increases energy expenditure and improves glucose intolerance through enhancing mitochondrial function in skeletal muscle of diabetic mice. J Biol Chem.

[13] Hurst JL (2009). Female recognition and assessment of males through scent. Behav Brain Res.

[14] Iannone A, Petroni A, Murru E, Cordeddu L, Carta G, Melis MP, Bergamini S, Casa LD, Cappiello L, Carissimi R (2009). Impairment of 8-iso-PGF(2ALPHA) isoprostane metabolism by dietary conjugated linoleic acid (CLA). Prostaglandins Leukot Essent Fatty Acids.

[15] Ishimoto T, Lanaspa MA, Le MT, Garcia GE, Diggle CP, Maclean PS, Jackman MR, Asipu A, Roncal-Jimenez CA, Kosugi T (2012). Opposing effects of fructokinase C and A isoforms on fructose-induced metabolic syndrome in mice. Proc Natl Acad Sci U S A.

[16] Kelley DS, Erickson KL (2003). Modulation of body composition and immune cell functions by conjugated linoleic acid in humans and animal models: benefits vs. risks. Lipids.

[17] Kim Y, Kim J, Whang KY, Park Y (2016). Impact of conjugated linoleic acid (CLA) on skeletal muscle metabolism. Lipids.

[18] Kiwaki K, Novak CM, Hsu DK, Liu FT, Levine JA (2007). Galectin-3 stimulates preadipocyte proliferation and is up-regulated in growing adipose tissue. Obesity.

[19] Kohjima M, Enjoji M, Higuchi N, Kato M, Kotoh K, Yoshimoto T, Fujino T, Yada M, Yada R, Harada N (2007). Re-evaluation of fatty acid metabolism-related gene expression in nonalcoholic fatty liver disease. Int J Mol Med.

[20] Lanaspa MA, Cicerchi C, Garcia G, Li N, Roncal-Jimenez CA, Rivard CJ, Hunter B, Andres-Hernando A, Ishimoto T, Sanchez-Lozada LG, et al. Counteracting roles of AMP deaminase and AMP kinase in the development of fatty liver. PloS one. 2012a;7:e48801. 10.1371/journal.pone.0048801 PMC3494720 23152807

[21] Lanaspa MA, Sanchez-Lozada LG, Choi YJ, Cicerchi C, Kanbay M, Roncal-Jimenez CA, Ishimoto T, Li N, Marek G, Duranay M, et al. Uric acid induces hepatic steatosis by generation of mitochondrial oxidative stress: potential role in fructose-dependent and -independent fatty liver. J Biol Chem. 2012b;287;40732-40744. 10.1074/jbc.M112.399899 PMC3504786 23035112

[22] Lanaspa MA, Sanchez-Lozada LG, Cicerchi C, Li N, Roncal-Jimenez CA, Ishimoto T, Le M, Garcia GE, Thomas JB, Rivard CJ, et al. Uric acid stimulates fructokinase and accelerates fructose metabolism in the development of fatty liver. PloS one. 2012c;7:e47948. 10.1371/journal.pone.0047948 PMC3480441 23112875

[23] Moya-Camarena SY, Vanden Heuvel JP, Blanchard SG, Leesnitzer LA, Belury MA (1999). Conjugated linoleic acid is a potent naturally occurring ligand and activator of PPARalpha. J Lipid Res.

[24] Nagao K, Inoue N, Wang YM, Shirouchi B, Yanagita T (2005). Dietary conjugated linoleic acid alleviates nonalcoholic fatty liver disease in Zucker (fa/fa) rats. J Nutr.

[25] Oh TS, Kwon EY, Choi JW, Choi MS, Yun JW (2011). Time-dependent hepatic proteome analysis in lean and diet-induced obese mice. J Microbiol Biotechnol.

[26] Park Y, Storkson JM, Albright KJ, Liu W, Pariza MW (1999). Evidence that the trans-10, cis-12 isomer of conjugated linoleic acid induces body composition changes in mice. Lipids.

[27] Piras A, Carta G, Murru E, Lopes PA, Martins SV, Prates JA, Banni S (2015). Effects of dietary CLA on n-3 HUFA score and N-acylethanolamides biosynthesis in the liver of obese Zucker rats. Prostaglandins Leukot Essent Fatty Acids.

[28] Reddy JK, Hashimoto T (2001). Peroxisomal beta-oxidation and peroxisome proliferator-activated receptor alpha: an adaptive metabolic system. Annu Rev Nutr.

[29] Saggerson ED, Greenbaum AL (1969). The effect of dietary and hormonal conditions on the activities of glycolytic enzymes in rat epididymal adipose tissue. Biochem J.

[30] Shaw PH, Held WA, Hastie ND (1983). The gene family for major urinary proteins: expression in several secretory tissues of the mouse. Cell.

[31] Shoji H, Deltour L, Nakamura T, Tajbakhsh S, Poirier F (2009). Expression pattern and role of galectin1 during early mouse myogenesis. Dev Growth Differ.

[32] Tan NS, Shaw NS, Vinckenbosch N, Liu P, Yasmin R, Desvergne B, Wahli W, Noy N (2002). Selective cooperation between fatty acid binding proteins and peroxisome proliferator-activated receptors in regulating transcription. Mol Cell Biol..

[33] Tirindelli R, Dibattista M, Pifferi S, Menini A (2009). From pheromones to behavior. Physiol Rev.

[34] Yang ZH, Miyahara H, Iwasaki Y, Takeo J, Katayama M (2013). Dietary supplementation with long-chain monounsaturated fatty acids attenuates obesity-related metabolic dysfunction and increases expression of PPAR gamma in adipose tissue in type 2 diabetic KK-Ay mice. Nutr Metab.

[35] Zhou Y, Jiang L, Rui L (2009). Identification of MUP1 as a regulator for glucose and lipid metabolism in mice. J Biol Chem.

